# Neuronavigation in falcine meningiomasare surgery: Initial results from a Vietnamese multi-center study

**DOI:** 10.1016/j.amsu.2022.104905

**Published:** 2022-11-21

**Authors:** Phuong Xuan Nguyen, Ha Thi-Ngoc Doan, Hoe Van Vu

**Affiliations:** a Department of Neurosurgery, Military Hospital 103, Hanoi, 12108, Viet Nam; b Bach Mai Hospital, Hanoi, 100000, Viet Nam

**Keywords:** Falcine meningiomasare, Neuronavigation, Neurosurgery, Surgical complications

## Abstract

**Background:**

Neuronavigation has been applied in neurocenters to help neurosurgeons plan the way to resect tumor totally and decline the neuro-injuries. This study aims to initially evaluate the result of microsurgery applying neuronavigation in treating falcine meningioma, which has not been reported comprehensively in Vietnam.

**Materials and methods:**

This cross-sectional study enrolled 62 patients diagnosised with falcine meningioma and treated by applying neuronavigation in operation in Neurosurgery Department of Military Hospital 103 and Viet Duc University Hospital from July 2015 to March 2017. Patients were assessed complications as well as residual lesions. Evaluate surgical results when discharging from hospital according to Ojemann MD standard.

**Results:**

Mean tumor volume was 67.37 ± 66.61 cm³ (range 6–370 cm³). Unilateral access site was the most common with 85.48%. Mean surgery duration was 209.84 ± 70.86 min. There were 27 out of 62 patients (43.55%) need blood transfusion in surgery. Tumor volume had an impact on blood transfusion in operation with p = 0.002. 82.26% of patients had total resection (Simpson I,II). There were 4.84% of patients having haemorrhage and 3 patients (4.84%) had wound infection after operation. Short-term outcome was evaluated according to Ojemann MD standard: good outcome was 67.74%, medium 17.74%, and poor outcome 14.52%.

**Conclusions:**

The application of neuronavigation and microsurgery enables surgeons to access falcine meningiomasare exactly, lessen the surgery duration, limit blood transfusion and complications after operation.

## Highlights

• Neuronavigation helps resect tumors totally and decline the neuro-injuries.
• Neuronavigation combining microsurgery can access falcine meningiomasare exactly.
• Neuronavigation helps microsurgery lessen the surgery duration, limit blood transfusion and complications after operation.
• Mean surgery duration was 209.84 ± 70.86 min, good outcome was 67.74%.

## 1 Introduction

Falcine meningioma amounts to 8% of intracranial meningioma [1]. According to Harvey William Cushing: falcine meningioma arises from the falx cerebri and is not totally related to superior sagittal sinus and cortex. It can grow in one or both hemispheres and in some cases, rises into inferior sagittal sinus [[2], [3], [4]]. These slow-growing and discretion tumors usually locate deeply, adjacently to the mid-line, between two hemispheres. Therefore, clinical symptoms of falcine meningioma are often hidden until presenting as a result of the huge tumor pressing on nearby tissue. This may hamper surgery, the main treatment of falcine meningioma, from early performance. Furthermore, the percentage of recurrence after surgery in patients with falcine meningioma is higher than in other locations, possibly due to subtotal resection [3,5].

Neuronavigation, a technique has been applied in neurocenters in Vietnam in recent years, is an assisting device to help neurosurgeons plan the way to resect tumor totally and decline the neuro-injuries. With deep falcine meningioma rising from anterior to posteior of falx, applying navigation will significantly enhance the operative manipulations and its results, particularly the appropriate removal of tumors. This study aims to initially evaluate the result of microsurgery applying neuronavigation in treating falcine meningioma, which has not been reported comprehensively in Vietnam.

## 2 Material and methods

Our cross-sectional study has been reported in line with the STROCSS criteria [6], with a Research Registry UIN: researchregistry7846.

This study enrolled 62 patients diagnosised with falcine meningioma and treated by applying neuronavigation in operation in Neurosurgery Department of Military Hospital 103 and Viet Duc University Hospital from July 2015 to March 2017.

### 2.1 Participants

The inclusion criteria was patients diagnosed with falcine meningioma based on clinical symptoms and diagnostic imaging of tumor characteristics; Pathological results are meningioma.

The exclusion criteria were as the followings: The patients were diagnosed with falcine meningioma but did not undergo operation because they and their family disagreed or the patients had combined diseases that did not allow to perform surgery; The patients experienced surgery without neuronavigation; Pathological result was not meningioma; The patients did not cooperate and follow reexamination postoperation; Falcine meningioma invaded superior sagittal sinus.

### 2.2 Surgical procedures

#### 2.2.1 Preparation of patients

Patients are pre-operatively assessed general status, clinical characteristics, diagnostic imaging, exact tumor location, size of tumor, relationship between tumor and skull, nearby brain structures and blood supply.

Falcine meningioma can result in surrounding edema and rise along white matter causing abnormalities in brain structures, which leads to increasing intracranial pressure (ICP). Therefore, a high dose of steroid preoperatively plays a crucial role in decreasing ICP. Quick transfusion of manitol was performed within 15 min with a dose of 1–2 g/kg when incising skin. High-frequency ventilation was maintained during operation.

### 2.3 Surgical technique

Patients are under general anesthesia. Head is on Mayfield skull clamp (manufactured by Codman, USA); at a higher position than heart.

Patients are placed in a position in which the center of tumor is the highest point. For falcine meningioma in frontal third and middle third which is adjacent to one third anterior, patients are in a supine position with head slightly lifted up. For falcine meningioma in middle third adjacent to one third posterior or in posterior third, patients are in a prone position with head lifted and sloped contralaterally with tumor.

**Fig. 1:**
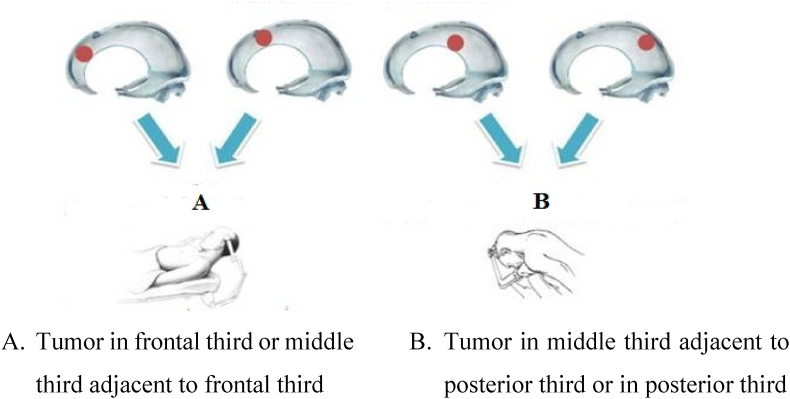
Surgical position.

Source: Murone D et al. (2017) [2].

**Fig. 2:**
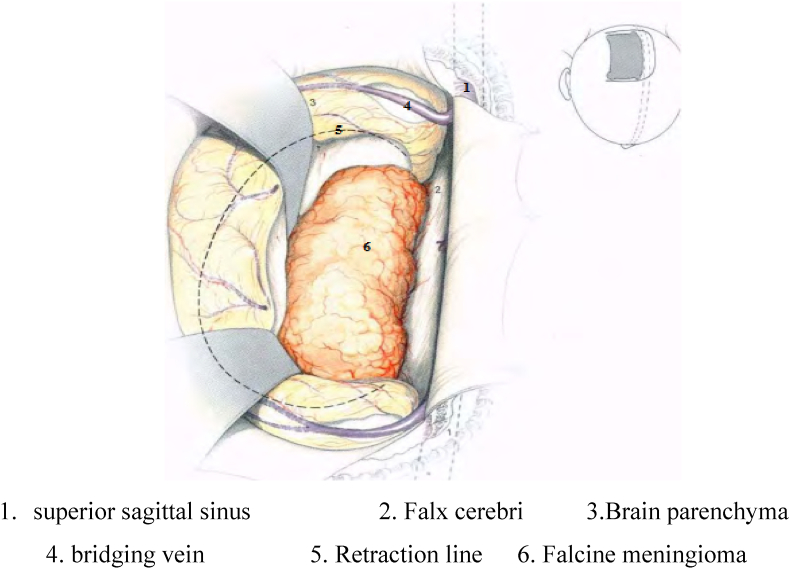
Related anatomy when removing frontal and middle third tumor.

source: Kenichiro S (1985) [7].

**Fig. 3:**
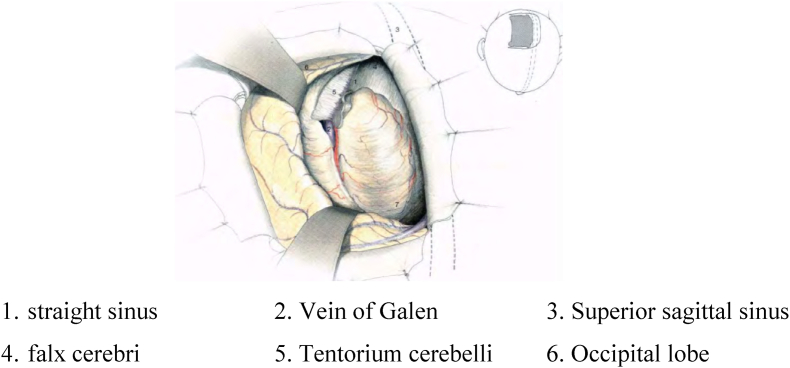
Related anatomy when removing posterior third tumor.

Source: Kenichiro S (1985) [7].

Using neuronavigator to detect location and margin of tumor (see Fig. 1). Planning the shortest way to resect tumor, limit neurological injuries, avoid damaging superior sagittal sinus and bridging vein in brain surface (Fig. 2) (see Fig. 3).

The incision for anterior third tumor is often an arc opening anteriorly or to the side of tumor. For middle third tumor, incision is horseshoe-shaped. With posterior third tumor, incision is a horseshoe-shaped facing down. Opening skull near the mid-line 1.5–2 cm laterally, on one or both sides of mid-line.

Evaluating the level of total resection based on classification of Simpson D. (1957) [8].

Grade I: complete removal, including basilar tumor and underlying bone and associated dura.

Grade II: complete removal and coagulation of dura attachment.

Grade III: complete removal without coagulation of dura attachment.

Grade IV: subtotal resection.

Grade V: decompression with or without biopsy.

Cranial dura mater is excised a 1 cm around the tumor under navigation to the venous sinuses and avoiding damaging sinuses and bridging veins.

Swinging brain to access longitudinal fissure and detect limit of tumor. Burn and control blood supply. After incising blood supply and basilar part of tumor, open the envelop and remove tumor. This is called depression inside tumor, which avoids swinging the brain tissue maximally. Intra-envelope resection of tumor is performed with ultrasonic knife. Cyst then is separated carefully from arachnoid and adhered blood vessels. Excising parts that covered tumor envelop partly when total resection is done.

For tumor rising bilaterally to mid-line, cutting blood supply, depressing and disecting tumor capsule can be performed bilaterally or unilaterally if part that arises contralateraly is small. Disecting and removing tumor are done by double microscope with high pecision, maximally preserve normal brain tissues and avoid swinging brain tissues, which results in damage in bridging vein.

In the process of resecting tumor, capsule must be well disected from vessels. especially callosal artery, pericallosal artery and deep veins. If these vessels are adhesive to the tumor, we do not make an effort for totally removal to avoid damages on the vessels. A cotton sponge was placed on the separation surface in order to prevent the peripheral cerebral cortex from being injured. Removing all the dural parts which are invaded to limit recurrence.

Dural plasty was performed after total resection. Material used is often autograft like galea aponeurotica or fascia latae sometimes is artificial dura, which are patched to avoid CSF leakage. Skull piece is ralaid and bone skull is fixing by screw.

### 2.4 Post-operation

Patients are under vital sign monitoring. First day after surgery, patients should conducted CT Scans to detect if there is an intracranila haemorrhage, gas or edema.

Most of patients are treated with dexamethazone 48 h preoperation or longer based of the edema. Postoperation, dexamathazone is continued within 5 days.

Patients are followed in terms of the bleeding status, epilepsy, new paralysis or worse paralysis and edema after surgery. Wound status, wound infection and meningitis should be detected immediately. Sutures were removed after 7–9 days of surgery.

Evaluate surgical results when discharging from hospital according to Ojemann MD standard [4].

## 3 Results

Ratio male/female was 1/3.77, mean age was 53,21 ± 12.01, range 24–76. The most common admission complaints were headache, and vomit, making up 43.55%. The rate of paralysis was 22.58%, of epilepsy was 16.13%, of mental disorders was 3.23%, of head trauma was 3.23% and of consciousness disorders was 1.61%.

Falcine meningioma in millde third location was the most popular with 46.77%, tumors on right hemisphere accounted for 40.32%, left hemisphere 40.32%, and bilateral tumor 19.36% (Table 1).

**Table 1:** Admission reasons and tumor location.

Admission complaints	Tumor location			Total (n (%))
	Frontal third	Middle third	Postal third	
	(n)	(n)	(n)	
Consciousness disorders	1	0	0	1 (1.61)
Headache, nausea	11	10	6	27 (43.55)
Movement paralysis	1	11	2	14 (22.58)
Sensory disorders	0	1	3	4 (6.45)
Epilepsy	2	7	1	10 (16.13)
Vision disorders	0	0	2	2 (3.23)
Mental disorders	2	0	0	2 (3.23)
Head trauma	0	0	2	2 (3.23)
Total (n (%))	17 (27.42)	29 (46.77)	16 (25.81)	62 (100)

Mean tumor volume was 67.37 ± 66.61 cm³ with the smallest being 6 cm³ and the largest being 370 cm³. CT Scans images: falcine meningioma often had a clear boudary 86.67%), hyperdense (86.67%), homogenous (62.22%), calcified tumor (22.22%), cystics tumor (4.44%) and vivid enhancement (93.33%) (Table 2).

**Table 2:** Images of falcine meningioma on CT Scans.

Imagines		Number of patients (n)	Rate (%)
Margin	Clear	39	86.67
	Unclear	6	13.33
Density	Hyperdense	39	86.67
	Hypodense	2	4.44
	Isodense	4	8.89
Homogenous	Yes	28	62.22
	No	17	37.78
Heterogenous	Cyst	2	4.44
	Calcification	10	22.22
	Necrosis	4	8.89
	Haemorrhage	1	2.22
Enhancement	Medium	3	6.67
	Vivid	42	93.33

Unilateral access site was the most common with 85.48%. The rate of bilateral tumor had unilateral access side was 33.33%. There were 33 out of 53 patients (62.26%) under bilateral cranial open and surgical duration between 120 and 240 min. For bilateral cranial opening and surgical duration over 240 min, there were 6 out of 9 patients (66.67%). Removal way was related to surgical duration with p = 0.013 (Table 3).

**Table 3:** Surgical duration and removal way of tumor.

Removal way	Surgical duration(minutes)					Total n(%)
	<120	120–180	180–240	240–300	>300	
	n(%)	n(%)	n(%)	n(%)	n(%)	
Unilateral	6(11.32)	18(33.96)	15(28.30)	8(15.10)	6(11.32)	53(100)
Bilateral	0	1(11.11)	2(22.22)	3(33.33)	3(33.34)	9(100)
p	0.013					

Mean surgery duration was 209.84 ± 70.86 min. There were 27 out of 62 patients (43.55%) need blood transfusion in surgery. Of those, 32.26% needed 2 units of blood transfusion. Tumor volume had an impact on blood transfusion in operation with p = 0.002 (Table 4).

**Table 4:** Relationship between amount of blood transfusion and tumor volume.

Amount of blood transfused	Tumor volume (cm³)			Total
	<50	50–100	>100	
	n (%)	n (%)	n (%)	
None	22 (70.97)	9 (52.94)	4 (28.57)	35
One unit	3 (9.68)	0	1(7.14)	4
Two units	5 (16.13)	8 (47.06)	7 (50)	20
Three units	1 (3.22)	0	2 (14.29)	3
Total	31 (100)	17 (100)	14 (100)	62
p	0.002			

82.26% of patients had total resection (Simpson I,II). Regarding complications, although there were 4.84% of patients having haemorrhage after operation, none of them needed resurgery since internal medicine brought good results. 3.23% of patients had edema while one patient (1.61%) had new paralysis and 3 patients (4.84%) had wound infection (Table 5). Short-term outcome was evaluated according to Ojemann MD standard: good outcome was 67.74%, medium outcome was 17.74%, and poor outcome was 14.52%. Comparing outcome with tumor volume, good outcome in group of tumor under 50 cm³ was 67.74%, and in group of tumor over 100 cm³ was 64.29% with p > 0.05.

**Table 5:** Complications postoperation.

Complications	Number of patients (n)	Rate (%)
Haemorrhage	3	4.84
Edema	2	3.23
New paralysis	1	1.61
Wound infection	3	4.84
Dead	0	0

## 4 Discussion

The proportion of contracting falcine meningioma in women is higher than in men. This supports the hypothesis that hormones result in the development of meningioma. Meningioma was reported to increase in size when the level of progesterone was higher in blood in pregnancy or luteal phase of menstrual cycle, especially with suprasellar meningioma. Progesterone receptors enable tumor to increase size due to augmenting blood supply, internal and external fluid as well as proliferation and developing tumor tissues as in pregnancy period. Factors causing the increasing of progesterone do have close relationship with surrounding edema in meningioma [9]. Based on this relationship, hormone therapy can be applied to manage meningioma. Developments of neurological symptoms can appear due to increasing size of tumor resulting from hormone released into blood vessels and creating posibility for edema.

The medium age was 53.21 ± 12.01 (range 24–76) years. This showed that the nature of falcine meningioma is slow-growing in years, and often asymptomatic. When the typical clinical symptoms appear, the tumor is already huge. This lead to many disadvantages in early diagnosis of falcine meningioma. In addition, limited indications for imaging diagnosis such as CT Scans or MRI can lead to ignorance of tumor.

In our study, there were 43.55% of patients having headache and nausea, resulting from increased ICP. Headache is the common symptom, but it is also the reason for late admission to the hospital. Headache is often mild, irregular, and easy to be mistaken with other diseases. Patients usually make an effort to deal with it or use internal medicine. Only when headache becomes worse and followed by epilepsy or movement paralysis, will patients be admitted to the hospital. Unfortunately, at this time, tumor is huge. As a result, early indications of CT Scans or MRI when patients admitted to hospital are essential.

According to Jo.K.W et al., of 154 patients having asymptomatic meningioma, falcien meningioma made up 19.5%. Tumor identified due to trauma was 8.4%, from health examination was 11.7%. The author followed 77 out of 154 patients and gave a conclusion that tumor size did not predictthe development of tumor. The calcified images on CT and age of patient would help evaluate the development of tumor and were evidence to choose appropriate surgical methods [10].

Falcine menigioma can develop unilaterally or bilaterally with or without falx pressing. In result of our study, falcine meningioma in middle third position is most common with 46.77%, followed by frontal third position with 27.42%, and postal third position accounted for the remaining 25.81%. In the studies of Giombini S, Pires A and Chung S. B, frontal third tumor had a higher percentage than other positions. Our research has consistent result with the research of Murrone D. The most common position is middle third, then the frontal third and postal third.

We approached tumor by 2 sites to open skull: unilateral and bilateral. There were 85.48% of patients receiving unilateral site. In groups of unilateral tumor, only one patients had bilateral site while in group of bilateral tumor, 33.33% of patient were conducted with unilateral sites. Hence, neuronavigation helps access tumor in minimum distance. In addition, microsurgery devices enable surgeons to open skull minimumly, to limit bleeding and damage nearby brain tissues. According to Zou F.X et al. [11], location, size of bilateral tumor and application of navigation in operation help surgeon identify exact site to remove tumor.

Zou F.S et al. (2012) and Das K.K (2017) suggested the approach method for falcine meningioma. For unilateral tumor, the best site is longitudinal fissure. For unilateral tumor with falx pressing, accessing tumor is achieved through contralateral longitudinal fissure. Bilateral tumor should be removed by bilateral sites [11,12].

Operation duration depends on many factors such as: location, size, density, devices and experience of surgeons. In our research, mean duration was 208 ± 70.17 min. The larger the tumor is, the longer the duration is with p < 0.01. Site for resection had a close relationship with duration, and removing tumor by bilateral site is longer than unilateral site, p = 0.013. Our result is similar to other authors around the world that neuronavigation enables surgeons to exactly, and directly access the tumor and lessen the surgery duration [13]. Kai Y et al. researched 42 patients undergoing preoperative embolization. Over 50% of tumors were supplied by segments of external carotid artery and embolization made tumor softer for an effective resection, and to reduce blood loss [14]. Therefore, the advantages of microsurrgery devices and application of neuronavigation in opearation significantly enhance the surgery duration.

There were 82.26% of tumors were completely resection (Simpson I,II), 17.8% was residual and none was decompression and biospy. There was no difference between total resection in frontal third, middle third and postal third tumor (p > 0.05). This is consistent with recommendations of authors that navigating in operation is influenced by edema, which creates difficulty for total resection [15,16].

Our result is similar to other authors’ that navigation helps remove tumor totally. However, total resection depends on competence experience of surgeons and the blood supply of tumors. Total resection is encouraged, but an effort to remove totally tumor leaving complications and impacts on operation result should not be performed [88]. Deep falcine meningioma, frontal third and middle third tumor are related to callosal artey, and pericallosal artery, which closely ahdere to tumor may result in difficulties for total resection. For middle third and postal third locations, tumor relating to superior, inferior sagittal sinuses, Vein of Galen, transverse vein or tumorwhich strongly adhering to falx are hard for total resection. In addition, calcified falx and firm tumor are factors preventing total resection [4].

There were 4.84% of bleeding, 3.23% of edema, but there was no patient under reopearation to coagulate or decompress. One patient had new paralysis and 3 (4.84%) had wound infection post operation. Murrone D et al. [2] after performing surgery on 95 patients with falcine meningioma, reported that 3 patients had worse paralysis and new numbness, deep coagulation happened in one patient, edema occurred in one patient not undergoing reoperation, and 2 patients had wound infection. In the research of Das K.K (2017) on 35 patiensts with falcine meningioma, 2 patients had bleeding, 1 patient had wound infection, 1 patient had deep coagulation and all patients did not experience reoperation. The author believed that bleeding may be caused by damages in callosal artery and pericallosal artery in unilateral tumor with falx pressing; in bilateral tumor in lower part of falx, tumor contiguous to superior sagittal sinus does cause bleeding [12]. Fotios K when performing microsurgery on 100 patients with parasagittal and falcine meningioma, found that there were 3 patients having new paralysis and one patient having venous infarction [17]. The appearance of falcine plexus increases the risk of bleeding during and postoperation [18].

No patient died in our study. According to research of Giombini S et al. on 127 patients experiencing microsurgery without navigation to remove parasagittal meingioma, dead rate was 13.3%. Most of these patients were in group Simpson I. The author believed that the main factors resulting in death were edema and venous damaging [19]. Hence, application of navigation declines complication during and postoperation.

Short-term outcome was evaluated when patients were discharged from hospital. Good outcome was 67.74%, medium was 17.74% and poor outcome was 14.52%. Comparing outcome with tumor volume, good outcome in group of tumor under 50 cm³ was 67.74%, and in group of tumor over 100 cm³ was 64.29% with p > 0.05. Thus, outcome was not related to tumor volume. According to Sade B et al. [20], outcome depends on pre-operation neurological status, age, location and size of tumor. In our study, patients with middle third tumor had good outcome of 58.62%, which is lower than in frontal third (82.35%) and postal third (68.75%). This may result from middle third location being near movement functional area which is sensitive with changes in brain tissues. In the study of Oyama H et al. (2012) [21] on 16 patients with meningioma, there were 12 patients with parasagittal meningioma and 4 patients with falcine meningioma in middle third location having movement paralysis and consciousness disorders before operation. After surgery, there were 11 patients having worse paralysis and consciousness compared preoperation. Author suggested surgeons should be prudent when performing operation in this location.

## 5 Conclusions

The application of neuronavigation and microsurgery enables surgeons to access falcine meningiomasare exactly, lessen the surgery duration, limit blood transfusion and complications after operation.

## Ethics approval and consent to participate

This study was approved by Viet Nam Military Medical University, Board of Directors of Miliraty Hospital 103 and Viet Duc University Hospital. All participants who took part in the study provided inform consent.

## Funding

None.

## Provenance and peer review

Not commissioned, externally peer-reviewed.

## Declaration of competing interest

The authors have declared that no competing interests exist.
